# Effect of Electronic Performance Monitoring on Employees’ Job Performance: A Social Information Processing Perspective

**DOI:** 10.3390/bs15030256

**Published:** 2025-02-23

**Authors:** Na Zhang, Xiang Sun, Chunhua Jin

**Affiliations:** School of Business, Beijing Information Science and Technology University, Beijing 102206, China; b15033@buaa.edu.cn (N.Z.); 2023020784@bistu.edu.cn (X.S.)

**Keywords:** electronic performance monitoring, job performance, self-efficacy

## Abstract

In the digital era, to enhance performance and achieve high-quality development, an increasing number of enterprises have begun to implement electronic performance monitoring. However, existing studies have yielded inconsistent conclusions regarding the impact of electronic performance monitoring on employees’ job performance. To clarify the overall relationship between these variables and explore the underlying causes of the discrepancies, this study, grounded in social information processing theory, examines the effects of two kinds of electronic performance monitoring (e.g., developmental and preventive) on employees’ job performance, as well as the mediating role of self-efficacy. On the basis of data collected at two time points from 223 employees in China, we test the mediation models. The findings indicate that both developmental and preventive electronic performance monitoring positively affect employees’ job performance, with self-efficacy serving as a partial mediator in this relationship. This study offers practical management strategies and establishes a foundation for the effective implementation of electronic performance monitoring in enterprises aimed at enhancing employees’ self-efficacy and job performance.

## 1. Introduction

In the digital era, enterprises are confronted with a rapidly evolving business environment and unprecedented levels of market competition. To enhance operational efficiency and effectiveness and achieve high-quality development, a growing number of organizations are adopting electronic performance monitoring (EPM). Originating from the concept of “electronic work monitoring”, EPM refers to the computerized collection, storage, analysis, and reporting of employees’ production activities. It is a commonly employed technical method used to observe, record, and analyze information directly or indirectly related to employees’ job performance (Ravid et al., 2020). EPM is characterized by the use of electronic systems to detect or evaluate employees’ job performance (Aiello & Kolb, 1995, 1997). A core reason for the adoption of EPM by enterprises is to monitor and maintain organizational performance in real time, thereby ensuring the continuous optimization of employees’ performance and the long-term development of the organization (Armstrong & Booksx, 2006).

Job performance is a dynamic representation of an employee’s work input, behavior, processes, and outcomes. It distinctly reflects the goals and tasks that employees are capable of completing (Murphy, 1993). Performance evaluation in enterprises entails a thorough analysis of various dimensions, including objectives, behaviors, abilities, and outputs. By evaluating their job performance, enterprises can gain a deeper understanding of their employees’ contributions. This process also helps organizations formulate more effective performance management strategies. In recent years, the rise of telecommuting and digital transformation has led to the growing prevalence of EPM in organizational management. The introduction of EPM offers new perspectives and tools for performance evaluation systems, enhancing both the efficiency and accuracy of this critical process (Stanton & Weiss, 2000).

With the advancement of information technology and the widespread use of the Internet, an increasing number of large enterprises are able to monitor their employees more extensively and intensively at a lower cost (Ravid et al., 2023; Yao et al., 2024). Various forms of electronic performance monitoring (EPM) have been implemented across numerous industries, including low-complexity jobs such as call center operators and ride-hailing drivers, as well as high-complexity jobs such as financial industry service personnel and medical professionals (Ravid et al., 2020; Thiel et al., 2022). The Internet financial technology industry, as one of the sectors where EPM is widely applied (Cao, 2024; Ravid et al., 2023), also places a high emphasis on employee performance management.

However, existing studies on the effect of EPM on employees’ job performance yield inconsistent results. Some research suggests that EPM enhances employees’ job performance (Bartels & Nordstrom, 2012; Goomas & Ludwig, 2009; O’Donnell et al., 2012), whereas other studies indicate that EPM may negatively impact job performance (T. E. Becker & Marique, 2014; Mallo et al., 2010). Additionally, some scholars have reported that the use of EPM has no significant effect on employees’ job performance (Al-Rjoub et al., 2008; Stanton & Sarkar-Barney, 2003). Therefore, to clarify the relationship between EPM and job performance and explore the reasons for these discrepancies, it is essential to discuss, in detail, the impact of EPM on employees’ job performance in the Internet financial technology industry.

Social information processing (SIP) theory posits that an individual’s social environment and the consequences of prior actions furnish information that influences their perceptions, attitudes, and behaviors. The decision made by the individual to interpret and process this information is a critical factor in shaping subsequent behaviors (Pfeffer, 1978). Consequently, depending on how employees perceive the purpose and function of EPM, various types of EPM can have distinct effects on employees’ emotional attitudes (such as self-efficacy) (Wells et al., 2007). Self-efficacy refers to an individual’s confidence or belief in his or her ability to achieve goals in a specific area (Bandura, 1977). It influences the level of effort and perseverance that individuals exhibit when faced with challenges. Additionally, self-efficacy is a crucial factor that impacts work investment and enhances employees’ job performance (Bandura, 1977).

Based on SIP theory, this study examines the effects of various types of EPM perceived by employees on their job performance, as well as the mediating role of self-efficacy in this relationship. This study makes several significant contributions. First, we respond to the calls of several scholars to conduct a classification study on electronic performance monitoring and explore the distinct pathways and mechanisms through which they influence employees’ job performance (König, 2025; Peng, 2022; Ravid et al., 2023; Yao et al., 2024). Additionally, the perspective of SIP theory is utilized to clarify the impact of EPM on employees’ job performance, providing new insights into EPM research. Consequently, this study provides new insights into EPM by clarifying the logical relationships between EPM and employees’ job performance. This study provides a foundation for the effective implementation of electronic performance monitoring in enterprises aimed at enhancing employees’ self-efficacy and job performance.

## 2. Theory and Hypothesis

### 2.1. Social Information Processing Theory

SIP theory posits that social information within an employee’s environment can influence their attitudes, motivations, and behaviors. Moreover, each stage of information processing by employees, such as the information reception process, the information processing process, and the impact of information on individual behavior, is also affected by the social environment (Gurbin, 2015). SIP theory suggests that individuals acquire information from their environment. Interpersonal interactions at work, individual behaviors, expressed traits, and perceptions of work roles are all sources of social information (Chen, 2024; Pfeffer, 1978; Zalesny & Ford, 1991). After obtaining this information, individuals encode, interpret, and process it and then respond accordingly (Zalesny & Ford, 1991). Zalesny and Ford proposed that the process of individual information processing includes learning, attribution, and judgment. Different ways of processing information could lead to different attitudes and outcomes (Pfeffer, 1978).

The core tenets of SIP theory are twofold: (1) The social environment in which an individual is situated contains various types of information that can influence their attitudes, motivations, and behaviors. People process this information to help them assess their work environment more accurately (Pfeffer, 1978). (2) When facing complex and uncertain social environments, people tend to rely more on information from the social environment to regulate their work attitudes, motivations, and behaviors (Pfeffer, 1978; Yam et al., 2018).

### 2.2. EPM and Job Performance

An organization’s EPM system prioritizes the monitoring of employees’ performance as its primary objective, which can significantly influence job performance (König, 2025; Ravid et al., 2023; Thiel et al., 2022). Job performance is a dynamic representation of an employee’s work input, behavior, processes, and outcomes. It distinctly reflects the goals and tasks that employees are capable of completing (Murphy, 1993). In their study, Borman and Motowidlo identified two dimensions of employee job performance: task performance and contextual performance (Borman & Motowidlo, 1993). These two perspectives have been acknowledged by scholars and are widely utilized in related research.

Task performance centers on the employee’s proficiency level in executing assigned tasks, which pertains specifically to their job responsibilities and is classified as in-role performance (Schmidt, 1986). In contrast, contextual performance highlights the importance of positive working relationships in motivating employees to execute tasks more effectively. This type of performance encompasses behaviors that contribute to organizational development, extending beyond the confines of the employee’s designated role (Scotter, 1996).

According to SIP theory, an individual’s activities are shaped by the intricate external social environment. The perceptions, attitudes, and behaviors of individuals are developed on the basis of the information they acquire. Furthermore, the interpretation and processing of this information significantly influence subsequent behaviors (Pfeffer, 1978). Based on employees’ purposes, EPM can be categorized into two types: developmental and preventive (Peng, 2022; Wells et al., 2007).

The purpose of developmental electronic performance monitoring (DEPM) is to facilitate employee development and to provide fair and meaningful performance feedback (McNall & Roch, 2009; Ravid et al., 2023). This monitoring method provides objective performance evaluations through detailed data and offers the necessary resources to support their work (Gilman et al., 2020). Moreover, quantitative performance feedback helps them improve their knowledge, skills, and abilities and increases their work resources (Ravid et al., 2023; Yao et al., 2024). Sewell posited that DEPM positively influences employees’ job performance by offering targeted training, enhancing their work capabilities, and ensuring equitable performance evaluations (Sewell, 2006). Additionally, Jeske and Santuzzi noted that DEPM can deliver focused training opportunities for employees, thereby fostering their learning and development (Jeske & Santuzzi, 2015). Furthermore, DEPM has the potential to increase employees’ trust in the organization, enhance their overall well-being, and ultimately improve their job performance.

Some scholars argue that electronic performance monitoring may bring out certain negative impacts. For example, it may reduce employees’ affective commitment to the organization (Jiang et al., 2020) and trigger anxiety among employees (W. J. Becker et al., 2021). Preventive electronic performance monitoring (PEPM) refers to the practice whereby an enterprise monitors its employees to deter them from engaging in behaviors that may be detrimental to the organization (Wells et al., 2007). Schleifer et al. posited that the implementation of EPM influences employees’ work behavior and attitudes throughout the work process, potentially resulting in adverse effects on their job performance (Schleifer et al., 1996). Bhave further argued that EPM has fostered a “panoramic prison” work environment, wherein employees are observed from various directions and angles during their work activities. Their processes and communications are recorded and are accessible for managerial review at any time (Bhave, 2014). Consequently, this pervasive surveillance exacerbates employees’ work-related stress and adversely impacts their performance (Ravid et al., 2023). Yao also suggested that PEPM imposes significant psychological pressure on employees, leading to a perception of personal resource loss. In such a context, employees tend to engage in self-protection behaviors, reducing their emotional and behavioral investment. This is particularly evident in complex innovative activities, where employees may become more cautious and less willing to invest time and effort (Yao et al., 2024). We propose the following hypotheses:

**Hypothesis** **1a.** *DEPM has a positive effect on employees’ task performance.*

**Hypothesis** **1b.** *DEPM has a positive effect on employees’ contextual performance.*

**Hypothesis** **2a.** *PEPM has a negative effect on employees’ task performance.*

**Hypothesis** **2b.** *PEPM has a negative effect on employees’ contextual performance.*

### 2.3. Mediating Role of Self-Efficacy

In the workplace, EPM is regarded as a systematic and continuous approach to evaluating employee performance (Stanton & Weiss, 2000). SIP theory suggests that employees’ attitudes and behaviors are shaped by their selection, processing, and interpretation of perceived information (Simon et al., 2019). Self-efficacy refers to an individual’s confidence in their ability to complete specific work tasks (Bandura, 1977). When employees perceive EPM as developmental, based on their understanding of the information, their self-efficacy may increase. Conversely, when employees view EPM as preventive, informed by their interpretation of the information, they may feel over-monitored and distrustful (Wells et al., 2007). In such cases, employees may experience a decline in self-efficacy.

Self-efficacy is a crucial factor in ensuring that individuals achieve high job performance (Bandura, 1989). Numerous studies have highlighted the significance of self-efficacy in enhancing job performance. Wood and Bandura suggested that self-efficacy primarily influences an individual’s choice process, cognitive process, motivation process, and emotional responses, thereby stimulating potential and improving job performance (Wood & Bandura, 1989). Stajkovic and Luthans reported a positive correlation between self-efficacy and task performance (Stajkovic & Luthans, 1998). Barling and Beattie noted that individuals with higher self-efficacy tend to exhibit greater task performance (Barling & Beattie, 2008). Kappagoda’s study involving 176 banking managers and 357 non-managerial personnel revealed that self-efficacy significantly positively affects employees’ task performance (Kappagoda, 2018). Lee’s research with a sample of 282 students indicated that self-efficacy influences their contextual performance (S. Lee, 2020). Additionally, Lee studied 212 hotel service employees and reported a positive correlation between self-efficacy and employees’ contextual performance (I.-G. Lee, 2021).

SIP theory posits that the external environment is replete with diverse information. Individuals’ interpretations of this information significantly influence their attitudes and behaviors (Pfeffer, 1978). When employees perceive EPM as developmental, their understanding of the information allows them to form and enhance their self-efficacy through the observation and imitation of positive feedback and behavioral patterns derived from monitoring. This belief drives them to work more diligently, set goals, engage in self-regulated behaviors, and undertake active learning and development initiatives, thereby enhancing their job performance (Seijtssupa & Gary, 2011). When employees perceive EPM as a preventive measure, on the basis of their interpretation of the information, they may feel over-monitored and distrusted (Wells et al., 2007). This perception can lead to a decline in their self-efficacy, ultimately resulting in decreased job performance (Hanks & Beier, 2012). Therefore, we propose the following hypotheses:

**Hypothesis** **3.** *Self-efficacy has a mediating role in the effect of DEPM on employees’ task performance.*

**Hypothesis** **4.** *Self-efficacy has a mediating role in the effect of DEPM on employees’ contextual performance.*

**Hypothesis** **5.** *Self-efficacy has a mediating role in the effect of PEPM on employees’ task performance.*

**Hypothesis** **6.** *Self-efficacy has a mediating role in the effect of PEPM on employees’ contextual performance.*

**Figure 1:**
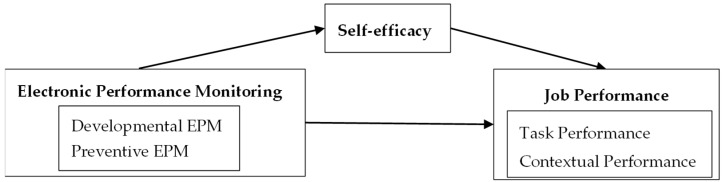
Theoretical model.

The model proposed in this paper is illustrated in Figure 1.

## 3. Materials and Methods

### 3.1. Participants and Procedure

As suggested by Gorsuch (1983), the ratio of the sample size to the number of items should be 5:1. Accordingly, this study included 44 items, necessitating a minimum of 130 samples (26 × 5). Given that structural equation modeling (SEM) requires a larger sample size (Schumacker & Lomax, 2010) and considering China’s substantial population, we ultimately issued 300 questionnaires.

This study was performed among Chinese employees of the Internet financial technology industry. These employees are engaged in diverse roles, including customer service and support, where they handle inquiries and resolve issues related to online transactions; risk management, focusing on credit assessment and compliance monitoring; technical development, involving software engineering and cybersecurity; data analysis, which includes mining large datasets to inform business strategies; and sales and marketing, aimed at promoting products and expanding market reach. Their tasks range from routine interactions with customers to complex problem solving and compliance monitoring.

The two-stage questionnaire survey was conducted online over a period of three months, from April to June 2024. During this period, the third author, with the assistance of the enterprise manager, distributed the survey questionnaires via the Credamo platform to employees. Credamo is a professional platform for specialized data collection in China. The first page of the survey outlined the study’s aims, potential risks, and benefits. It was made clear to the employees that the survey was conducted solely for academic purposes. Participation was voluntary, and no personal data were collected. All questionnaires were completed in the room by the participants and subsequently collected by the third author upon completion.

The specific steps were as follows: The questionnaire survey at the first stage included items measuring electronic performance monitoring and demographic information. Additionally, the participants were required to provide the last four digits of their mobile phone numbers at the end of the questionnaire. A total of 260 valid questionnaires were collected at this stage. During the second stage, the same group of participants received a second questionnaire, which included items measuring self-efficacy, job performance, and demographic information. The participants were again required to provide the last four digits of their mobile phone numbers. A total of 240 valid questionnaires were collected at this stage. After cross-referencing the last four digits of mobile phone numbers and demographic characteristics, invalid questionnaires (such as those with random answers, missing values, or overly patterned responses) were excluded. Finally, 223 valid questionnaires were obtained, with an effective response rate of 74.3%. The demographics of the valid sample are shown in Table 1.

### 3.2. Measures

All scales (Appendix A) in this paper use a maturity scale and a 5-point Likert scale (from 1 “completely disagree” to 5 “completely agree”).

EPM: Employees reported EPM at the first stage via the scale developed by Wells et al. (2007) and translated into Chinese by Peng (2022). It involved two dimensions: DEPM and PEPM. Each dimension had three items, for a total of six items. Examples include “The company uses the EPM to help me complete the work better” and “The company uses the EPM to prevent possible misconduct among employees at work”. The Cronbach α coefficients were 0.862 and 0.841, respectively.

Self-efficacy: Self-efficacy was measured at the second stage via the GSES scale developed by Schwarzer et al. (1999) and translated into Chinese by Wang (2001). The scale initially included 20 items but was then reduced to 10 items. Examples include “For me, it is easy to stick to my ideals and achieve my goals” and “At work, if I put in the necessary effort, I will be able to solve most problems”. The Cronbach α coefficient of this scale was 0.925.

Job performance: Employees reported their job performance at the second stage via the scale developed by Borman and Motowidlo (1993) and translated into Chinese by scholar Yu (1996). There were five items for each dimension. Examples include “My work efficiency is very high” or “I often cooperate with other colleagues in the company”. The Cronbach α coefficients were 0.852 and 0.871, respectively.

### 3.3. Data Analysis

Data analysis was conducted via SPSS 27.0 and Mplus 8.0. The specific statistical procedures employed were as follows: First, SPSS 27.0 was used to conduct the common method bias test. Second, confirmatory factor analysis (CFA) was performed via Mplus 8.0. Third, SPSS 27.0 was again utilized for validity analysis, descriptive statistical analysis, and correlation analysis. Finally, structural equation modeling (SEM) was applied to test the hypotheses via Mplus 8.0.

## 4. Results

### 4.1. Common Method Variance Testing

In the editing and distribution of the questionnaire, this study emphasized the anonymity of the questionnaire and scrambled the order of the items. The aim was to avoid the single thinking of the subjects in the process of completing the questionnaire and to control common method variance. Harman’s single-factor test was used to control for common method variance. The variance explained by the first principal component analysis was 37.268% (less than 40%), indicating that the common method variance in this research fell within an acceptable threshold (Hao & Lirong, 2004).

### 4.2. Confirmatory Factor Analysis

CFA was performed to ensure that all the variables in our study had satisfactory discriminant validity. As shown in Table 2, compared with the other models, the five-factor model (DEPM, PEPM, self-efficacy, task performance, and contextual performance) had the best fit (χ^2^ = 297.284, χ^2^/df = 1.029, RMSEA = 0.011, CFI = 0.997, TLI = 0.997, SRMR = 0.046) and was better than the other models.

### 4.3. Validity Analysis

This paper used SPSS 27.0 to verify its validity, mainly from the following perspectives.

Content validity: The scales used in this paper are all derived from mature scale systems in existing studies and have been used for research and validation many times in previous studies. Thus, their content validity meets the requirements of the research.

This study used the mean square extraction value (AVE value) and the combination reliability (CR) value to analyze the convergent validity.

Convergent validity: The KMO test was performed via SPSS 27.0. The KMO value of this scale was 0.938. Moreover, the Bartlett test was performed via SPSS 27.0, and the result was *p* = 0.001 < 0.05. Thus, these findings confirm that the data met the conditions for factor analysis. This study used the mean square extraction value (AVE value) and the combination reliability (CR) value to analyze the convergent validity.

Table 3 shows that the AVE values of the variables of the questionnaire used in this paper are all above 0.5, and the combined reliability (CR) values are also distributed above 0.7. This means that the data obtained in this study have good convergent validity.

Discriminant validity: According to Table 4, the value on the diagonal in the table is greater than any value in the column where it is located. Thus, the discriminant validity of the data is determined to be satisfactory. Based on the abovementioned checks of the convergent validity and content validity, the data validity is confirmed.

### 4.4. Correlation Analysis

To test the proposed hypotheses, a correlation analysis was performed on DEPM, PEPM, self-efficacy, task performance, and contextual performance. As shown in Table 5, DEPM was positively correlated with PEPM. There was a positive correlation between task performance and contextual performance (r = 0.388, *p* < 0.01). Self-efficacy was positively correlated with DEPM (r = 0.424, *p* < 0.01) and PEPM (r = 0.439, *p* < 0.01). Task performance was positively correlated with DEPM (r = 0.313, *p* < 0.01), PEPM (r = 0.340, *p* < 0.01), and self-efficacy (r = 0.386, *p* < 0.01). Contextual performance was positively correlated with DEPM (r = 0.411, *p* < 0.01), PEPM (r = 0.443, *p* < 0.01), and self-efficacy (r = 0.444, *p* < 0.01).

### 4.5. Hypothesis Testing

**Figure 2:**
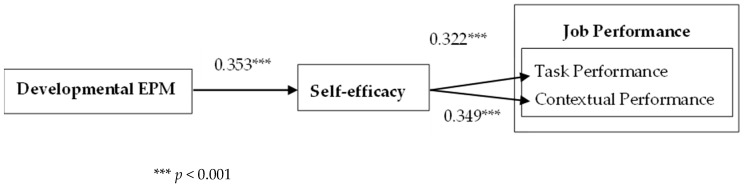
The effect of DEPM on job performance.

We first tested the effect of DEPM on employees’ job performance. After controlling for all the control variables, DEPM had a significant positive effect on employees’ task performance (b = 0.158, SE = 0.062, *p* < 0.05) and contextual performance (b = 0.240, SE = 0.057, *p* < 0.001). Thus, H1a and H1b were supported. The results of SEM are shown in Figure 2. DEPM positively affected self-efficacy (b = 0.353, SE = 0.048, *p* < 0.001). Self-efficacy positively affected task performance (b = 0.322, SE = 0.073, *p* < 0.001) and contextual performance (b = 0.349, SE = 0.067, *p* < 0.001).

In addition, the bootstrap method was used to calculate the 95% confidence interval of the mediating effect to test the mediating effect. The number of repeated samples was set to 5000. The results show that DEPM had a significant indirect effect on employees’ task performance through self-efficacy (b = 0.114, SE = 0.030, *p* < 0.001, 95% CI = (0.063, 0.180)), and the confidence interval did not include 0. DEPM had a significant indirect effect on employees’ contextual performance through self-efficacy (b = 0.123, SE = 0.030, *p* < 0.001, 95% CI = (0.074, 0.191)), and the confidence interval did not include 0. Thus, H3 and H4 were supported.

**Figure 3:**
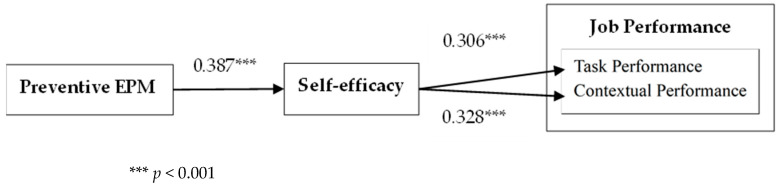
The effect of PEPM on job performance Results of multilevel path analysis.

We then tested the effect of PEPM on employees’ job performance. The results show that PEPM had a significant positive effect on employees’ task performance (b = 0.193, SE = 0.065, *p* < 0.01) and contextual performance (b = 0.287, SE = 0.062, *p* < 0.001). Thus, both H2a and H2b were not supported. The SEM results are shown in Figure 3. PEPM positively affected self-efficacy (b = 0.387, SE = 0.050, *p* < 0.001). Self-efficacy positively affected task performance (b = 0.306, SE = 0.071, *p* < 0.001) and contextual performance (b = 0.328, SE = 0.069, *p* < 0.001).

In addition, the bootstrap method was used to calculate the 95% confidence interval of the mediating effect to test the mediating effect. The number of repeated samples was set to 5000. The results show that PEPM had a significant indirect effect on employees’ task performance through self-efficacy (b = 0.118, SE = 0.031, *p* < 0.001, 95% CI = (0.065, 0.190)), and the confidence interval did not exclude 0. PEPM had a significant indirect effect on employees’ contextual performance through self-efficacy (b = 0.127, SE = 0.031, *p* < 0.001, 95% CI = (0.074, 0.195)), and the confidence interval did not include 0. Thus, H5 and H6 were supported.

## 5. Discussion

Based on SIP theory, this study explored the different influencing mechanisms of DEPM/PEPM on employees’ job performance in the Internet financial technology industry and the mediating role of self-efficacy. The following results were obtained.

First, DEPM positively influences employees’ job performance. This finding suggests that as employees perceive a higher level of DEPM, their job performance is more likely to improve. SIP theory posits that individuals acquire information from their environment, and after obtaining this information, they encode, interpret, and process it, subsequently responding appropriately (Pfeffer, 1978; Zalesny & Ford, 1991). Additionally, some scholars’ research suggests that employees’ perceptions of the purpose of developmental electronic performance monitoring can enhance job satisfaction and employee creativity, ultimately improving work performance (Ahmed et al., 2022; Ravid et al., 2023; Siegel et al., 2022). This finding also aligns with the study conducted by McNall and Roch (2009), who reported that when employees recognize a developmental purpose, they feel valued by their managers. This perception enhances employees’ sense of interpersonal and informational fairness, which can in turn strengthen their trust in the organization and lead to improved job performance. A study conducted by Jeske and Santuzzi (2015) further confirmed the positive impact of DEPM on employees’ job performance. They reported that DEPM facilitates targeted training for employees, thereby promoting their learning and development. Additionally, it enhances employees’ trust in the organization, improves their overall well-being, and contributes to an increase in job performance.

Our findings indicate that self-efficacy serves as a partial mediator between DEPM and employees’ job performance. Specifically, a higher degree of employees’ perception of DEPM is correlated with increased self-efficacy, which in turn enhances job performance. This result aligns with the conclusions drawn in the majority of existing empirical studies (Bhave, 2014; Jeske & Santuzzi, 2015; Kappagoda, 2018; McNall & Roch, 2009; Sadri, 2011).

Second, PEPM positively influences employees’ self-efficacy and job performance. This finding suggests that a higher level of PEPM perceived by employees facilitates the enhancement of their job performance and self-efficacy. SIP theory suggests that different ways of processing information can lead to varying attitudes and outcomes (Pfeffer, 1978). Based on SIP theory, the positive influence of PEPM on self-efficacy and job performance can be explained by several factors. First, this may be attributed to PEPM serving as a deterrent method aimed at preventing unfavorable behaviors within the organization (Jeske & Santuzzi, 2015). For example, monitoring Internet usage and blocking non-work-related websites can help mitigate cyberloafing (Tomczak et al., 2017). Additionally, it encourages employees to focus on work-related tasks and diminishes the occurrence of counterproductive behaviors (Bhave, 2014), thereby enhancing their self-efficacy and overall job performance.

Then, this may also be attributed to the fact that, for workers, whether EPM is overt or covert, D’Urso noted (in agreement with Foucault) that “the perception that a person may be being monitored, even if it is not actually occurring, can be a powerful tool for management and can have serious potential impacts on individuals” (D’Urso, 2006). In other words, the mere existence of monitoring may enhance employees’ sense of responsibility toward their work and intensify the competitive environment (Tomczak et al., 2017). This perception may serve as a motivation for them to improve the quality of their work and their overall job performance.

Furthermore, employees in high-stress positions, such as those in the Internet finance industry, who frequently face customer complaints and excessive demands, may positively evaluate electronic monitoring as a means to manage their challenging work environment and seek organizational support (Ravid et al., 2023). Therefore, contrary to common sense, the more exposed these employees are to customer “attacks”, the more likely they are to accept not only DEPM but also PEPM. By positively perceiving the purpose of EPM, employees are more likely to enhance their self-efficacy.

In addition, this study was conducted in China, Chinese organizations often emphasize collective harmony and hierarchical structures, which can shape employees’ perceptions of EPM (Esmaelzadeh et al., 2017; Hofstede, 1984). Under such a cultural background, employees are more inclined to accept organizational management decisions, including the implementation of PEPM. This acceptance stems from a focus on the overall interests of the organization and trust in the intentions of the management. Moreover, the traditional Chinese cultural concept of “face” also affects employees’ reactions to EPM to some extent (Ma, 2018; Oetzel et al., 2001; Ravid et al., 2023). Employees may be more concerned about how to maintain a good image and reputation within the organization, and the preventive monitoring system is seen as a tool that helps them avoid mistakes and preserve their personal and organizational reputation (Peng, 2022). Therefore, employees may be more willing to accept this kind of monitoring to ensure that their behavior meets the organization’s expectations and standards.

Our findings also indicate that self-efficacy plays a partial mediating role between PEPM and employees’ job performance. This suggests that a higher degree of employees’ perception of PEPM correlates with improvements in both self-efficacy and job performance. This effect may stem from the original intention of PEPM, which is to prevent employee misconduct (Wells et al., 2007). When the purpose of monitoring is open and transparent, employees are likely to perceive it as the organization’s commitment to fairness and transparency, thereby fostering trust and a sense of equity (McNall & Roch, 2009). This sense of fairness and trust motivates employees to increase their self-efficacy (Ng & Lucianetti, 2016), thereby improving their job performance (Judge & Bono, 2001; McDonald & Siegall, 2001; Sadri, 2011). Furthermore, the essence of PEPM is still to monitor employees’ performance, recording and providing employees’ performance data in real time (Peng, 2022; Ravid et al., 2023). This timely feedback helps employees make self-adjustments in their work, enhancing their confidence in their abilities, thereby improving self-efficacy and boosting their performance (Jeske & Santuzzi, 2015; Wells et al., 2007).

### 5.1. Theoretical Implications

Our findings in the present study contribute to the literature in several ways.

First, this study refines and enriches the classification of EPM. While most existing research has concentrated on the impact of EPM on employees’ job performance, there has been comparatively little discussion regarding how different types of EPM influence this performance. This study differentiates between DEPM and PEPM (Wells et al., 2007), providing a detailed analysis of their distinct effects on employees’ job performance. It also responded to the calls of several scholars to conduct a classification study on electronic performance monitoring (Peng, 2022; Ravid et al., 2023; Yao et al., 2024). This research not only enhances the theoretical understanding of EPM but also offers a new perspective on how various EPM strategies can impact employees’ job performance.

Second, the perspective of SIP theory is introduced to elucidate the impact of EPM on employees’ job performance, which offers fresh insights into the research on EPM. Existing research predominantly employs theories such as planned behavior theory (Abraham et al., 2019), social exchange theory (Ahmed et al., 2022; Peng, 2022), and social facilitation theory (Claypoole & Szalma, 2019; Thompson et al., 2009) to explore the connections between employees’ perceptions of EPM and their work attitudes, behaviors, and outputs. SIP theory posits that employees’ interpretation and processing of information within an organization significantly influence their attitudes and behavioral responses (Pfeffer, 1978). Consequently, this study lays a new foundation for understanding the relationships between EPM and employees’ work attitudes, behaviors, and outputs.

Finally, in the process of studying the influencing mechanisms of EPM on employees’ job performance, most previous studies utilized fairness (Cropanzano et al., 2001), trust (Ahmed et al., 2022), or psychological contracts (Peng, 2022) as mediating variables. Introducing self-efficacy as a mediating variable offers a novel theoretical perspective for understanding how EPM impacts employees’ job performance and uncovers a new associative mechanism between EPM and employees’ job performance. Self-efficacy, defined as an individual’s belief in their abilities (Bandura, 1977), can elucidate the behavioral motivations and emotional responses of employees when confronted with EPM, thereby influencing their job performance. The integration of self-efficacy into the research framework of EPM underscores the importance of considering the psychological experiences of employees in the implementation of digital management tools. This study contributes to elucidating the internal psychological mechanisms through which EPM affects employees’ job performance and provides theoretical support for fostering a more humane and efficient work environment.

### 5.2. Management Implications

In the digital business era, enterprises must transcend traditional monitoring concepts and view EPM as a tool to foster employee development rather than merely a mechanism for supervision and evaluation. We aim to transform this technology into a positive support system that assists employees in enhancing their job performance while achieving both personal and professional growth.

First, enterprises should fully leverage the advantages of DEPM to enhance employees’ confidence and enthusiasm. They can reinforce the positive feedback mechanism to ensure that feedback is specific, actionable, and timely (McNall & Roch, 2009). Thus, employees can obtain development and improve their performance. Furthermore, enterprises can focus on positive reinforcement by highlighting what employees are doing well and recognizing and rewarding achievements publicly to boost morale and self-efficacy (Ravid et al., 2023). This positive motivation can increase employees’ self-efficacy, thereby improving their job performance.

Second, when implementing monitoring, enterprises must respect the privacy of employees. They should avoid invading personal space, minimize employees’ feelings of insecurity, and mitigate the potential negative effects of PEPM (Wells et al., 2007). Additionally, enterprises can inform employees in advance about the purpose of monitoring to avoid feelings of insecurity and psychological burden (Ravid et al., 2023). By balancing the motivational aspects of DEPM with the restrictive elements of PEPM, enterprises can foster a work environment that encourages personal growth among employees. This approach not only enhances organizational efficiency but also facilitates the mutually beneficial development of both employees and the organization.

Finally, leadership support is a critical factor in enhancing employee self-efficacy, which in turn drives better job performance and overall organizational success (Bandura, 1989). A positive organizational culture is a powerful driver of employee self-efficacy, influencing how employees perceive their abilities and their confidence in achieving goals (Bandura, 2002; Ribeiro, 2024). Through these measures, employees’ self-efficacy can be enhanced, which in turn improves their job performance. Thus, it can assist organizations in achieving the ultimate goal of fostering growth and development for both the organization and its employees.

### 5.3. Limitations and Future Research

This study examined the impact of EPM on employees’ job performance. However, future research could expand the scope to include other performance types, such as innovation performance. Such investigations would enhance our understanding of the multidimensional effects of EPM. Additionally, while this research focuses primarily on individual employee performance, there remains a lack of analysis regarding its influence at the team level. Future studies should explore how EPM affects team dynamics, including its potential impact on collaboration, innovation capacity, and overall team performance. This multilevel and multidimensional approach would provide a more comprehensive understanding of the actual effects of EPM and offer more targeted management strategies and practical recommendations for organizations.

Second, when examining the effect of EPM on employees’ job performance, future research should also consider the influence of the emotional perspective in addition to the cognitive perspective of self-efficacy. Emotion, as a crucial component of employees’ work experience, significantly impacts job performance (Ngo, 2021). Future studies should extend beyond a singular cognitive perspective to comprehensively account for multiple factors, including emotional influences. Additionally, exploring how EPM affects employees’ emotional experiences and how these experiences, in turn, impact job performance can enrich the literature. By adopting this multidimensional research approach, organizations can develop more effective management strategies to enhance both the job performance and the overall well-being of their employees.

Finally, when examining the effect of EPM on employees’ job performance, it is essential to consider how the varying natures of work and diverse organizational climates influence employees’ perceptions of EPM, subsequently affecting their job performance. Different job characteristics necessitate distinct work behaviors and performance standards (Li et al., 2016), whereas the organizational climate offers employees a psychological and emotional backdrop for their work (Cullen et al., 2003). Therefore, future research should explore the specific impact of EPM on employees’ job performance within various work and organizational climates. Such studies will contribute to a more nuanced and comprehensive understanding of the mechanisms underlying EPM. Additionally, this research can inform more effective management strategies aimed at enhancing employees’ job performance and overall development.

## 6. Conclusions

The findings indicate that both developmental and preventive electronic performance monitoring have a positive effect on employees’ job performance, with self-efficacy serving as a partial mediator in this relationship. This study offers practical management strategies and establishes a foundation for the effective implementation of electronic performance monitoring in enterprises aimed at enhancing employees’ self-efficacy and their job performance.

## Figures and Tables

**Table 1:** Demographic characteristics.

Demographics	Classification	Frequency	Percentage
Gender	Male	119	53.4%
	Female	104	46.6%
Education	Specialized and below	124	55.61%
	Undergraduate	81	36.32%
	Master’s and above	18	8.07%
Job nature	General staff	114	51.12%
	Technical staff	72	32.29%
	First-line manager	19	8.52%
	Middle or senior manager	6	2.69%
	Others	12	5.38%
Years of tenure	1 year and below	22	9.87%
	Between 1 and 3 years	76	34.08%
	Between 3 and 5 years	87	39.01%
	5 years and above	38	17.04%

Note: *N* = 223.

**Table 2:** Results of the confirmatory factor analysis.

Fit Indicators	χ^2^	χ^2^/df	RMSEA	SRMR	CFI	TLI
Five-factor model	297.284	1.029	0.011	0.046	0.997	0.997
Four-factor model	656.558	2.241	0.075	0.074	0.899	0.877
Three-factor model	657.286	2.221	0.074	0.075	0.890	0.879
Two-factor model	1060.459	3.559	0.107	0.102	0.768	0.747
One-factor model	1661.112	5.556	0.143	0.123	0.585	0.549

Note: One-factor model: DEPM + PEPM + SE + TP + CP; two-factor model: DEPM + PEPM + SE, TP + CP; three-factor model: DEPM + PEPM, SE, TP + CP; four-factor model: DEPM, PEPM, SE, TP + CP; five-factor model: DEPM, PEPM, SE, TP, CP. DEPM = developmental electronic performance monitoring, PEPM = preventive electronic performance monitoring, SE = self-efficacy, TP = task performance, CP = contextual performance.

**Table 3:** Results of convergent validity testing.

Variables	AVE Value	CR Value
DEPM	0.664	0.856
PEPM	0.623	0.832
Self-efficacy	0.548	0.916
Task performance	0.576	0.871
Contextual performance	0.571	0.868

**Table 4:** Results of discriminant validity testing.

Variables	DEPM	PEPM	Self-Efficacy	Task Performance	Contextual Performance
DEPM	0.680				
PEPM	0.848	0.718			
Self-efficacy	0.424	0.439	0.547		
Task performance	0.313	0.340	0.386	0.446	
Contextual performance	0.411	0.443	0.444	0.388	0.543

**Table 5:** Means, standard deviations, and correlations of the variables.

Variables	M	SD	1	2	3	4	5
1. DEPM	3.046	1.238					
2. PEPM	3.118	1.170	0.848 **				
3. Self-efficacy	3.117	1.031	0.424 **	0.439 **			
4. Task performance	3.271	1.073	0.313 **	0.340 **	0.386 **		
5. Contextual performance	3.045	1.093	0.411 **	0.443 **	0.444 **	0.388 **	

Note: *N* = 223, ** *p* < 0.05 (2-tailed).

## Data Availability

The raw data supporting the conclusions of this article will be made available by the authors on request.

## Appendix A

**Electronic performance monitoring**

1. The company uses electronic performance monitoring to help me perform my job better.
2. The company uses electronic performance monitoring to produce examples of correct procedures that can be used to train others.
3. The company uses the electronic performance monitoring to point out areas of my performance that need improvement.
4. The company uses the electronic performance monitoring to prevent wrongdoing on the part of [company] employees.
5. The company uses the electronic performance monitoring to detect possible misconduct or fraud.
6. The company uses the electronic performance monitoring to discourage employees from doing something wrong.

**Self-efficacy**

1. I can always manage to solve difficult problems if I try hard enough.
2. If someone opposes me, I can find means and ways to get what I want.
3. It is easy for me to stick to my aims and accomplish my goals.
4. I am confident that I could deal efficiently with unexpected events.
5. Thanks to my resourcefulness, I know how to handle unforeseen situations.
6. I can solve most problems if I invest the necessary effort.
7. I can remain calm when facing difficulties because I can rely on my coping abilities.
8. When I am confronted with a problem, I can usually find several solutions.
9. If I am in trouble, I can usually think of something to do.
10. No matter what comes my way, I am usually able to handle it.

**Job performance**

1. My work efficiency is very high.
2. I can fulfill the duties described in the job description.
3. My work can meet the expected standards.
4. I can complete work tasks according to formal performance assessment requirements.
5. Overall, I can do well my job in my company.
6. I often take the initiative to solve problems at work.
7. I often take on extra work to help others or improve team performance.
8. I often cooperate with other colleagues in the company.
9. When colleagues encounter problems, I will offer support and encouragement.
10. Overall, I care deeply about my colleagues and the company.

## References

[B1-behavsci-15-00256] Abraham M., Niessen C., Schnabel C., Lorek K., Grimm V., Möslein K., Wrede M. (2019). Electronic monitoring at work: The role of attitudes, functions, and perceived control for the acceptance of tracking technologies. Human Resource Management Journal.

[B2-behavsci-15-00256] Ahmed F., Soomro S. A., Tunio F. H., Ding Y., Qureshi N. A. (2022). Performance monitoring, subordinate’s felt trust and ambidextrous behavior; toward a conceptual research framework. Frontiers in Psychology.

[B3-behavsci-15-00256] Aiello J. R., Kolb K. J. (1995). Electronic performance monitoring and social context: Impact on productivity and stress. Journal of Applied Psychology.

[B4-behavsci-15-00256] Aiello J. R., Kolb K. J. (1997). Computer-based performance monitoring and productivity in a multiple task environment. Journal of Business&Psychology.

[B5-behavsci-15-00256] Al-Rjoub H., Zabian A., Qawasmeh S. (2008). Electronic monitoring: The employees point of view. Journal of Social Sciences.

[B6-behavsci-15-00256] Armstrong M., Booksx I. (2006). Performance management: Key strategies and practical guidelines.

[B7-behavsci-15-00256] Bandura A. (1977). Self-efficacy: Toward a unifying theory of behavioral change. Psychological Review.

[B8-behavsci-15-00256] Bandura A. (1989). Human agency in social cognitive theory. American Psychologist.

[B9-behavsci-15-00256] Bandura A. (2002). Social cognitive theory in cultural context. Applied Psychology.

[B10-behavsci-15-00256] Barling J., Beattie R. (2008). Self-efficacy beliefs and sales performance. Journal of Organizational Behavior Management.

[B11-behavsci-15-00256] Bartels L. K., Nordstrom C. R. (2012). Examining big brother’s purpose for using electronic performance monitoring. Performance Improvement Quarterly.

[B12-behavsci-15-00256] Becker T. E., Marique G. (2014). Observer effects without demand characteristics: An inductive investigation of video monitoring and performance. Journal of Business and Psychology.

[B13-behavsci-15-00256] Becker W. J., Belkin L. Y., Conroy S. A., Tuskey S. E. (2021). Killing me softly: Organizational e-mail monitoring expectations’ impact on employee and significant other well-being. Journal of Management.

[B14-behavsci-15-00256] Bhave D. P. (2014). The invisible eye? Electronic performance monitoring and employee job performance. Personnel Psychology.

[B15-behavsci-15-00256] Borman W., Motowidlo S. (1993). Expanding the criterion domain to include elements of contextual performance.

[B16-behavsci-15-00256] Cao Y. (2024). The double-edged sword effect of electronic performance monitoring on employees’ responsible innovation behavior. Chinese Journal of Management.

[B17-behavsci-15-00256] Chen T. S. H. (2024). The double-edged effect of leader error reporting on employee cognitive evaluation: Based on social information processing theory. Human Resources Development of China.

[B18-behavsci-15-00256] Claypoole V. L., Szalma J. L. (2019). Electronic performance monitoring and sustained attention: Social facilitation for modern applications. Computers in Human Behavior.

[B19-behavsci-15-00256] Cropanzano R., Byrne Z. S., Bobocel D. R., Rupp D. E. (2001). Moral virtues, fairness heuristics, social entities, and other denizens of organizational justice. Journal of Vocational Behavior.

[B20-behavsci-15-00256] Cullen J. B., Parboteeah K. P., Victor B. (2003). The effects of ethical climates on organizational commitment: A two-study analysis. Journal of Business Ethics.

[B21-behavsci-15-00256] D’Urso S. C. (2006). Who’s watching us at work? Toward a structural? Perceptual model of electronic monitoring and surveillance in organizations. Communication Theory.

[B22-behavsci-15-00256] Esmaelzadeh F., Abbaszadeh A., Borhani F., Peyrovi H. (2017). Ethical sensitivity in nursing ethical leadership: A content analysis of Iranian nurses experiences. The Open Nursing Journal.

[B23-behavsci-15-00256] Gilman E., Castejón V. D. R., Loganimoce E., Chaloupka M. (2020). Capability of a pilot fisheries electronic monitoring system to meet scientific and compliance monitoring objectives. Marine Policy.

[B24-behavsci-15-00256] Goomas D. T., Ludwig T. D. (2009). Standardized goals and performance feedback aggregated beyond the work unit: Optimizing the use of engineered labor standards and electronic performance monitoring. Journal of Applied Social Psychology.

[B25-behavsci-15-00256] Gorsuch R. L. (1983). Three methods for analyzing limited time-series (N of 1) data. Behavioral Assessment.

[B26-behavsci-15-00256] Gurbin T. (2015). Enlivening the machinist perspective: Humanising the information processing theory with social and cultural influences. Procedia-Social and Behavioral Sciences.

[B27-behavsci-15-00256] Hanks A. R., Beier M. E. (2012). Differential prediction of preparatory and performance self-efficacy judgments. Human Performance.

[B28-behavsci-15-00256] Hao Z., Lirong L. (2004). Statistical remedies for common method biases. Advances in Psychological Science.

[B29-behavsci-15-00256] Hofstede G. (1984). Culture’s consequences: International differences in work-related values.

[B30-behavsci-15-00256] Jeske D., Santuzzi A. M. (2015). Monitoring what and how: Psychological implications of electronic performance monitoring. New Technology, Work and Employment.

[B31-behavsci-15-00256] Jiang H., Tsohou A., Siponen M., Li Y. (2020). Examining the side effects of organizational Internet monitoring on employees. Internet Research.

[B32-behavsci-15-00256] Judge T. A., Bono J. E. (2001). Relationship of core self-evaluations traits—Self-esteem, generalized self-efficacy, locus of control, and emotional stability—With job satisfaction and job performance: A meta-analysis. Journal of Applied Psychology.

[B33-behavsci-15-00256] Kappagoda S. (2018). Self-efficacy, task performance and contextual performance: A Sri Lankan experience. Journal of Human Resource and Sustainability Studies.

[B34-behavsci-15-00256] König C. J. (2025). Electronic monitoring at work. Annual Review of Organizational Psychology and Organizational Behavior.

[B35-behavsci-15-00256] Lee I.-G. (2021). The effects of internal marketing on self-efficacy, service orientation and contextual performance in the hotel industry in Korea. The Journal of International Trade & Commerce.

[B36-behavsci-15-00256] Lee S. (2020). The effects of AIS education on academic self-efficacy and contextual performance. The Journal of the Korea Contents Association.

[B37-behavsci-15-00256] Li A., Bagger J., Cropanzano R. (2016). The impact of stereotypes and supervisor perceptions of employee work–family conflict on job performance ratings. Human Relations.

[B38-behavsci-15-00256] Ma B. (2018). The u-shaped impact of perceived over-qualification on employee creativity: The mediation role of competence face stresss. Nankai Business Review.

[B39-behavsci-15-00256] Mallo J., Nordstrom C. R., Bartels L. K., Traxler A. (2010). The effect of age and task difficulty. Performance Improvement Quarterly.

[B40-behavsci-15-00256] McDonald T., Siegall M. (2001). The effects of technological self-efficacy and job focus on job performance, attitudes, and withdrawal behaviors. Journal of Psychology.

[B41-behavsci-15-00256] McNall L. A., Roch S. G. (2009). A social exchange model of employee reactions to electronic performance monitoring. Human Performance.

[B42-behavsci-15-00256] Murphy K. R. (1993). Personnel selection in organizations. The Academy of Management Review.

[B43-behavsci-15-00256] Ng T. W. H., Lucianetti L. (2016). Within-individual increases in innovative behavior and creative, persuasion, and change self-efficacy over time: A social–cognitive theory perspective. Journal of Applied Psychology.

[B44-behavsci-15-00256] Ngo T. T. (2021). Impact of psychological capital on job performance and job satisfaction: A case study in vietnam. Journal of Asian Finance, Economics and Business.

[B45-behavsci-15-00256] O’Donnell A. T., Ryan M. K., Jetten J. (2012). The hidden costs of surveillance for performance and helping behaviour. Group Processes & Intergroup Relations.

[B46-behavsci-15-00256] Oetzel J., Ting-Toomey S., Masumoto T., Yokochi Y., Pan X., Takai J., Wilcox R. (2001). Face and facework in conflict: A cross-cultural comparison of China, Germany, Japan, and the United States. Communication Monographs.

[B47-behavsci-15-00256] Peng Z. (2022). Exploring the influence of electronic performance monitoring on employee value co-creation in gig economy. Human Resources Development of China.

[B48-behavsci-15-00256] Pfeffer S. J. (1978). A social information processing approach to job attitudes and task design. Administrative Science Quarterly.

[B49-behavsci-15-00256] Ravid D. M., Tomczak D. L., White J. C., Behrend T. S. (2020). EPM 20/20: A review, framework, and research agenda for electronic performance monitoring. Journal of Management.

[B50-behavsci-15-00256] Ravid D. M., White J. C., Tomczak D. L., Miles A. F., Behrend T. S. (2023). A meta-analysis of the effects of electronic performance monitoring on work outcomes. Personnel Psychology.

[B51-behavsci-15-00256] Ribeiro S. (2024). Enhancing teachers’ self-efficacy supported by coaching in the content of open schooling for sustainability. Sustainability.

[B52-behavsci-15-00256] Sadri G. (2011). Boosting performance through self-efficacy. American Society for Training & Development.

[B53-behavsci-15-00256] Schleifer L. M., Galinsky T. L., Pan C. S. (1996). Mood disturbances and musculoskeletal discomfort: Effects of electronic performance monitoring under different levels of VDT data-entry performance. International Journal of Human Computer Interaction.

[B54-behavsci-15-00256] Schmidt F. L. (1986). Impact of job experience and ability on job knowledge, work sample performance, and supervisory ratings of job performance. Journal of Applied Psychology.

[B55-behavsci-15-00256] Schumacker R. E., Lomax R. G. (2010). A beginner’s guide to structural equation modeling.

[B56-behavsci-15-00256] Schwarzer R., Mueller J., Greenglass E. (1999). Assessment of general perceived self efficacy on the internet: Data collection in cyber-space. Anxiety, Stress, and Copying.

[B57-behavsci-15-00256] Scotter J. R. V. (1996). Interpersonal facilitation and job dedication as separate facets of contextual performance. Journal of Applied Psychology.

[B58-behavsci-15-00256] Seijtssupa G. H., Gary P. (2011). The effect of commitment to a learning goal, self-efficacy, and the interaction between learning goal difficulty and commitment on performance in a business simulation. Human Performance.

[B59-behavsci-15-00256] Sewell G. (2006). Coercion versus care: Using irony to make sense of organizational surveillance. Academy of Management Review.

[B60-behavsci-15-00256] Siegel R., König C. J., Lazar V. (2022). The impact of electronic monitoring on employees’ job satisfaction, stress, performance, and counterproductive work behavior: A meta-analysis. Computers in Human Behavior Reports.

[B61-behavsci-15-00256] Simon L. S., Bauer T. N., Erdogan B., Shepherd W. (2019). Built to last: Interactive effects of perceived overqualification and proactive personality on new employee adjustment. Personnel Psychology.

[B62-behavsci-15-00256] Stajkovic A. D., Luthans F. (1998). Social cognitive theory and self-efficacy: Goin beyond traditional motivational and behavioral approaches. Organizational Dynamics.

[B63-behavsci-15-00256] Stanton J. M., Sarkar-Barney S. T. M. (2003). A detailed analysis of task performance with and without computer monitoring. International Journal of Human-Computer Interaction.

[B64-behavsci-15-00256] Stanton J. M., Weiss E. M. (2000). Electronic monitoring in their own words: An exploratory study of employees’ experiences with new types of surveillance. Computers in Human Behavior.

[B65-behavsci-15-00256] Thiel C. E., Prince N. R., Sahatjian Z. (2022). The (electronic) walls between us: How employee monitoring undermines ethical leadership. Human Resource Management Journal.

[B66-behavsci-15-00256] Thompson L. F., Sebastianelli J., Murray N. P. (2009). Monitoring online training behaviors: Awareness of electronic surveillance hinders e-learners. Journal of Applied Social Psychology.

[B67-behavsci-15-00256] Tomczak D. L., Lanzo L. A., Aguinis H. (2017). Evidence-based recommendations for employee performance monitoring. Business Horizons.

[B68-behavsci-15-00256] Wang C. K. (2001). Evidences for reliability and validity of the chinese version of general self efficacy scale. Journal of Applied Psychology.

[B69-behavsci-15-00256] Wells D. L., Moorman R. H., Werner J. M. (2007). The impact of the perceived purpose of electronic performance monitoring on an array of attitudinal variables. Human Resource Development Quarterly.

[B70-behavsci-15-00256] Wood R., Bandura A. (1989). Social cognitive theory of organizational management. Academy of Management Review.

[B71-behavsci-15-00256] Yam K. C., Christian M. S., Wei W., Liao Z., Nai J. (2018). The mixed blessing of leader sense of humor: Examining costs and benefits. Academy of Management Journal.

[B72-behavsci-15-00256] Yao D., Li Y., Wu Y., Zhu P. (2024). Gain or loss? The double-edged sword effect of electronic performance monitoring on employees’ innovation behavior. Human Resources Development of China.

[B73-behavsci-15-00256] Yu D. (1996). The impact of quality management human face system factors on job performance. Doctor thesis.

[B74-behavsci-15-00256] Zalesny M. D., Ford J. K. (1991). Extending the social information processing perspective: New links to attitudes, behaviors, and perceptions. Organizational Behavior & Human Decision Processes.

